# Fighting the Fracture Cascade: Early and Repeated Balloon Kyphoplasty as a Bridge Until the Effects of Osteoporosis Treatment Become Apparent in a Super-Aged Patient

**DOI:** 10.7759/cureus.84419

**Published:** 2025-05-19

**Authors:** Tatsuya Tanaka, Xuan Liu, Hirotaka Shojima, Nobuaki Momozaki, Eiichiro Honda, Akira Matsuno

**Affiliations:** 1 Department of Neurosurgery, International University of Health and Welfare Narita Hospital, Narita, JPN; 2 Department of Neurosurgery, Shiroishi Kyoritsu Hospital, Shiroishi, JPN; 3 Department of Neurosurgery, Shojima Neurosurgery, Saga, JPN; 4 Department of Neurosurgery, Imari Arita Kyoritsu Hospital, Arita, JPN; 5 Department of Neurology, Shiroishi Kyoritsu Hospital, Shiroishi, JPN

**Keywords:** activities of daily living (adl), adjacent vertebral fracture, balloon kyphoplasty, early surgical intervention, fracture cascade, minimally invasive spine surgery, osteoporotic vertebral fracture, spinal alignment, super-aged patient, teriparatide

## Abstract

Osteoporotic vertebral compression fractures are common in the elderly and frequently lead to pain, spinal deformity, and decreased activities of daily living (ADL). While balloon kyphoplasty (BKP) is known to provide rapid pain relief and improve mobility, it does not prevent the occurrence of subsequent adjacent vertebral fractures, especially before the therapeutic effects of pharmacologic osteoporosis treatments such as teriparatide become apparent. We report the case of an 87-year-old super-aged female patient who experienced a cascade of five adjacent vertebral fractures within four months. After conservative treatment failed, she underwent BKP for L1 and L4, resulting in immediate pain relief. However, new fractures subsequently occurred at Th12, L2, and L3, each requiring further BKP. Early surgical intervention after each fracture successfully restored mobility and preserved independence in ADL. Over a five-year follow-up period, no new fractures were observed, and spinal alignment was maintained. This case highlights the utility of early and repeated BKP as a bridging strategy until the effects of osteoporosis treatment take hold. Timely, minimally invasive interventions can prevent deterioration of spinal alignment and preserve quality of life in super-aged patients with severe osteoporosis.

## Introduction

Osteoporotic vertebral compression fractures are commonly observed in elderly individuals and lead to severe low back pain and spinal deformity, which can result in a decline in activities of daily living (ADL) [1-3]. Conservative treatments, including bed rest and the use of a back brace, are typically considered first-line therapies. However, prolonged bed rest in elderly patients may increase the risk of disuse syndrome and complications, potentially causing a significant decline in ADL [4,5]. Balloon kyphoplasty (BKP) is a minimally invasive procedure for osteoporotic vertebral fractures, in which a balloon is inflated within the collapsed vertebral body to restore vertebral height, followed by the injection of bone cement to stabilize the fracture. BKP has been reported to provide rapid pain relief, facilitate early mobilization, maintain ADL, and promote social reintegration [6,7]. However, BKP serves only as a symptomatic treatment and does not address the underlying osteoporosis, which may lead to new vertebral fractures postoperatively. Among these, adjacent vertebral fractures are a significant concern. Risk factors for adjacent fractures include advanced age, the presence of pre-existing vertebral fractures, increased local kyphosis due to collapse, and the presence of intravertebral clefts [8-10]. Here, we present the case of an elderly patient with osteoporosis who experienced recurrent adjacent vertebral fractures within a short period but was able to maintain good ADL through repeated BKP procedures.

## Case presentation

**Figure 1:**
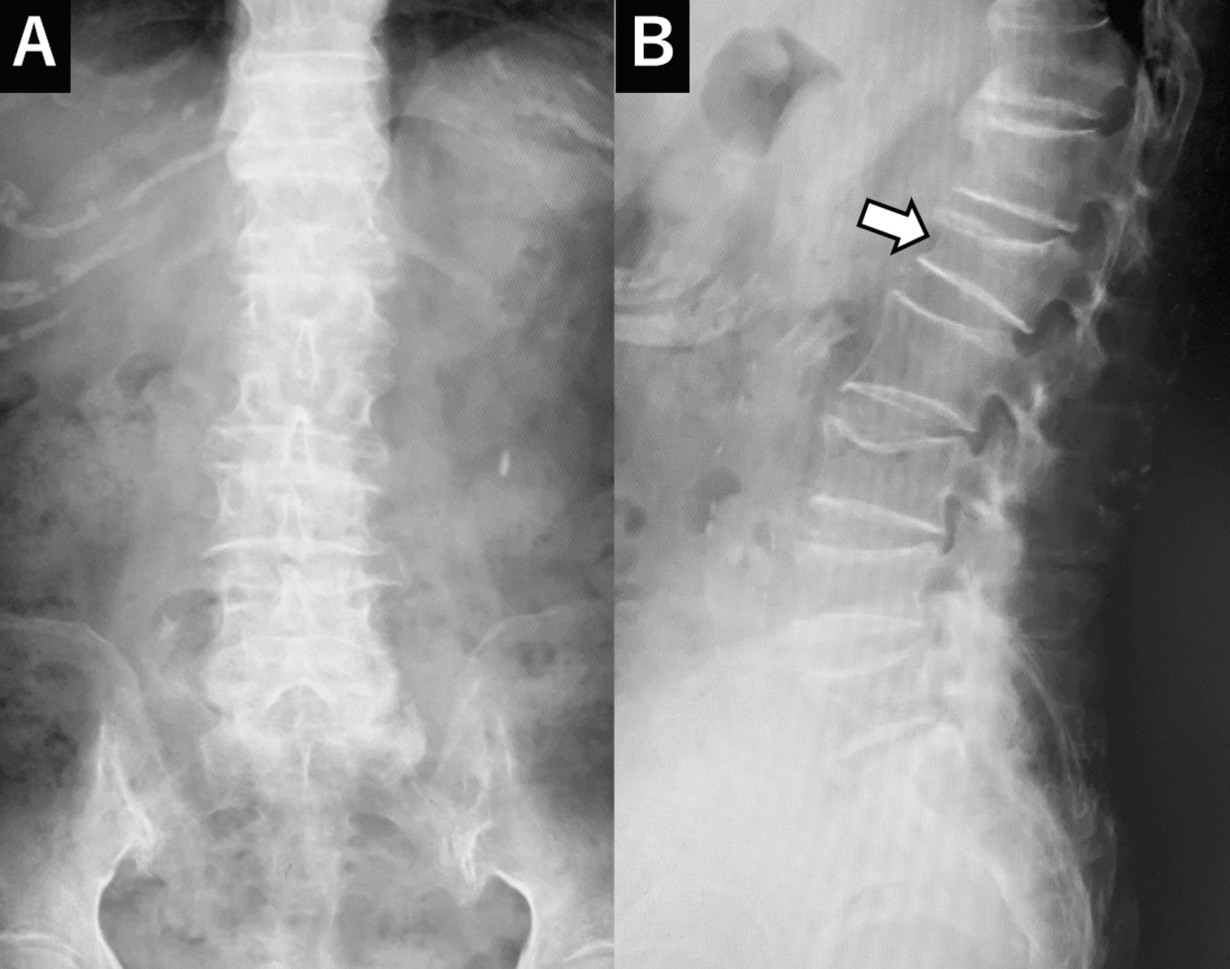
Initial X-ray An initial X-ray reveals a vertebral compression fracture at L1 (arrow).

**Figure 2:**
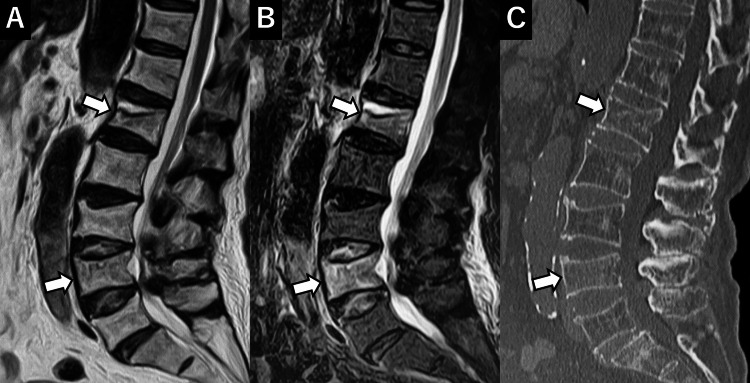
Initial MRI and CT A: T2-weighted image (T2WI), B: Short T1 inversion recovery (STIR), C: CT. New vertebral compression fractures are observed at L1 and L4 (arrows).

An 87-year-old woman with a medical history of diabetes, hypertension, and bilateral knee osteoarthritis presented with severe low back pain following an attempt to rise from a lying position at home. The pain resulted in immobility, and despite two weeks of persistent discomfort, the patient sought medical attention at a local clinic. Radiologic evaluation, including X-ray, MRI, and CT of the thoracolumbar spine, revealed fresh compression fractures at the first lumbar (L1) and fourth lumbar (L4) vertebrae (Figures 1, 2).

Conservative treatment, consisting of rest and corset fixation, was initiated. However, due to the severity of pain and significant functional limitations, she was referred to our department four weeks after symptom onset, at which time BKP was planned. Osteoporosis treatment was concurrently initiated with subcutaneous teriparatide injection, and she was encouraged to mobilize using a semi-rigid corset.

**Figure 3:**
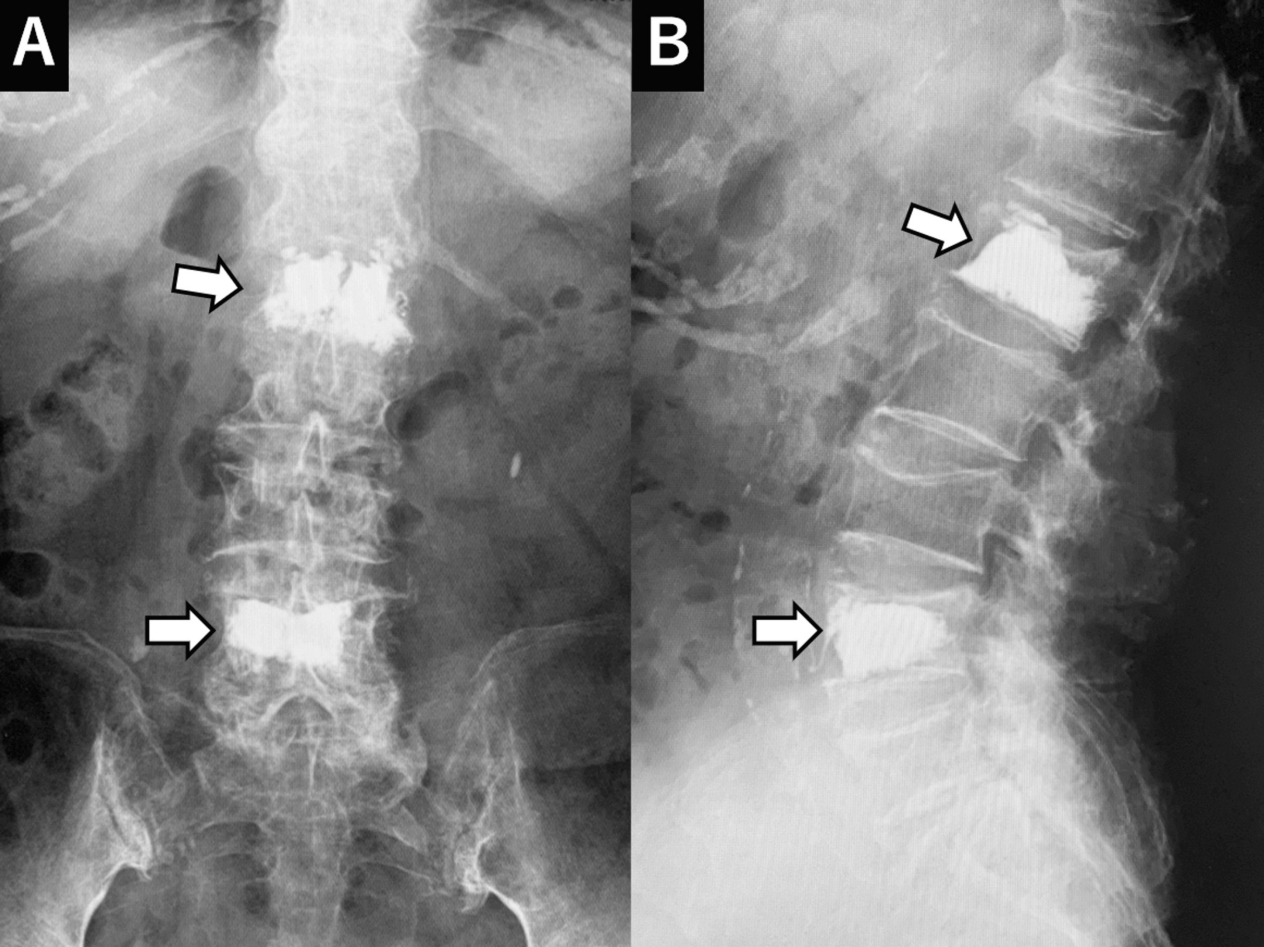
Post-first BKP X-ray An X-ray taken after the first balloon kyphoplasty (BKP) shows bone cement in L1 and L4 (arrows).

Approximately four weeks after the onset of symptoms (two weeks after initiating conservative treatment), percutaneous BKP was performed at L1 and L4. The procedure involved the insertion of bone access needles, consisting of cannula tubes and stylets, into the bilateral pedicles at the correct spinal levels (L1 and L4) under fluoroscopic guidance. A working tunnel was created, and balloons were inserted through the bilateral working tunnels to elevate and restore vertebral body height. Cement was subsequently injected. The procedure resulted in significant pain relief, allowing the patient to resume daily activities. There were no intraoperative complications, such as cement leakage, and immediately postoperatively, the patient's low back pain was markedly reduced, with restoration of L1 vertebral body height (Figure 3).

Postoperative recovery was uneventful, and she was mobilized promptly while wearing a semi-rigid corset.

**Figure 4:**
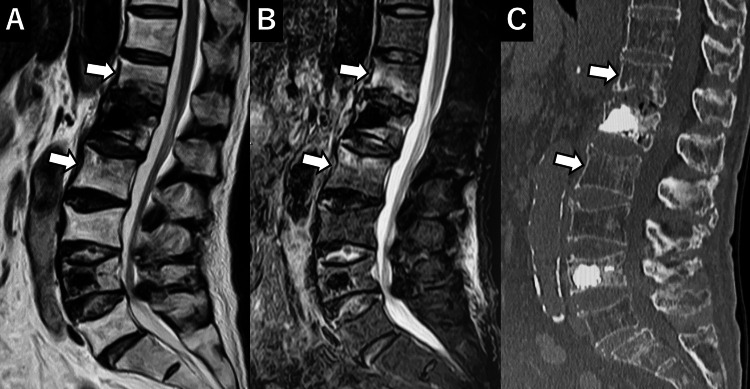
Pre-second surgery MRI and CT A: T2-weighted image (T2WI), B: Short T1 inversion recovery (STIR), C: CT. New vertebral compression fractures are noted at Th12 and L2 (arrows).

Two weeks after discharge, the patient experienced a recurrence of back pain. Imaging revealed a new compression fracture at the 12th thoracic vertebra (Th12). Conservative management was attempted, but approximately four weeks later, a new fracture at the second lumbar vertebra (L2) was identified, resulting in increased low back pain. Both fractures occurred at adjacent levels to the vertebrae treated during the initial surgery (Figure 4).

**Figure 5:**
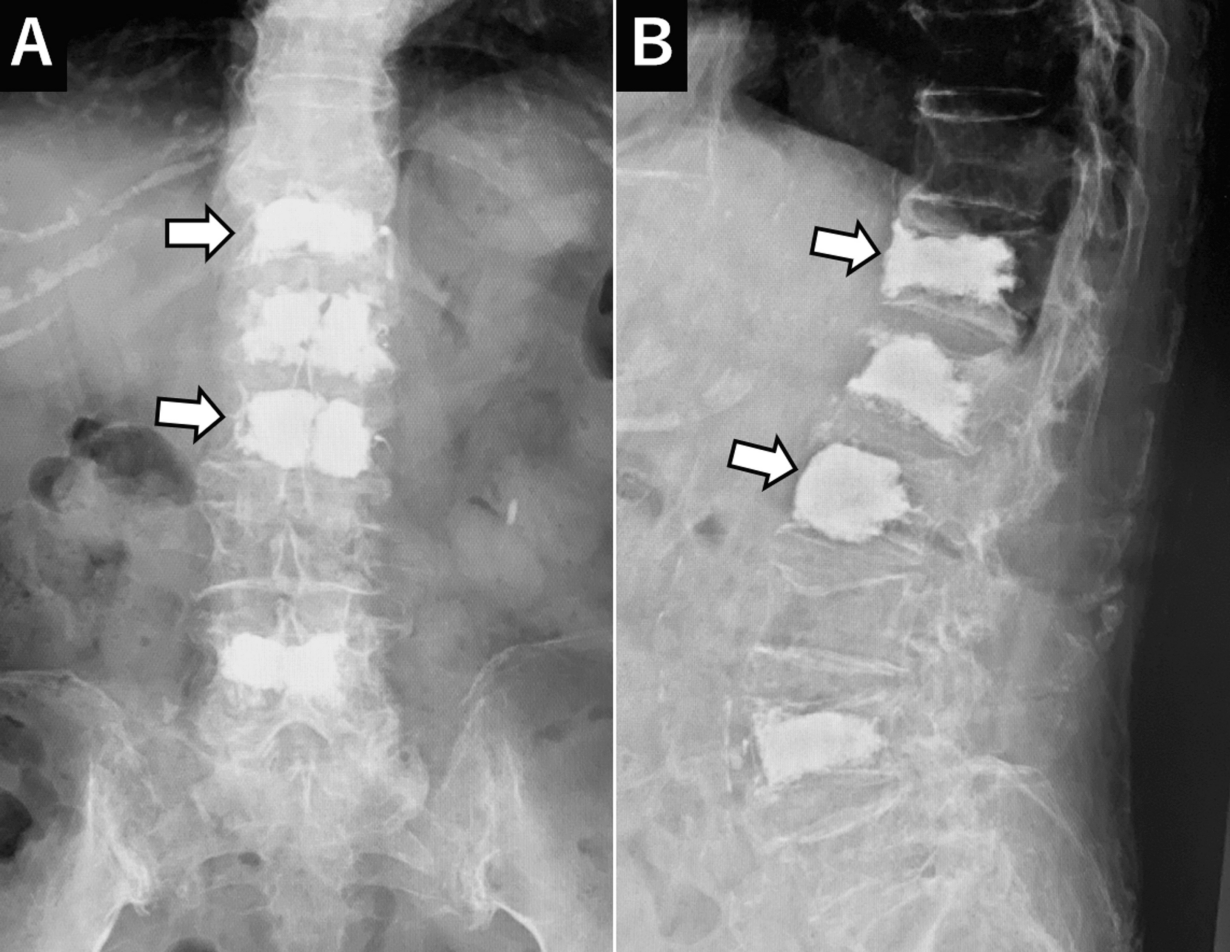
Post-second BKP X-ray An X-ray taken after the second balloon kyphoplasty (BKP) shows bone cement in Th12 and L2 (arrows).

The patient expressed a strong desire for early pain relief and continued independent living. Consequently, a second BKP procedure was performed six weeks after the initial surgery to address the Th12 and L2 compression fractures (Figure 5).

The technique and surgical procedure were identical to those used in the first surgery. No neurological complications or cement leakage occurred postoperatively, and pain relief was achieved immediately. The patient was discharged home a few days later.

**Figure 6:**
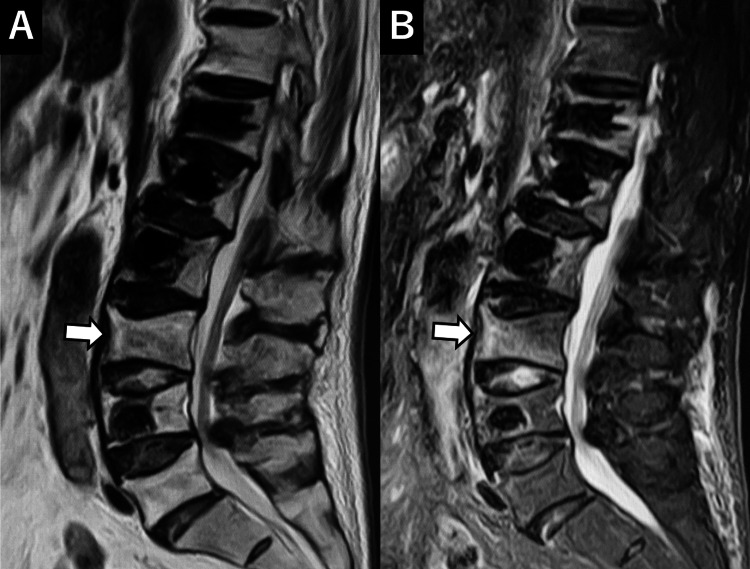
Pre-third surgery MRI A: T2-weighted image (T2WI), B: Short T1 inversion recovery (STIR). A new vertebral compression fracture is seen at L3 (arrow).

Two weeks after discharge, the patient developed a recurrence of low back pain due to a new compression fracture at the third lumbar vertebra (L3) (Figure 6).

**Figure 7:**
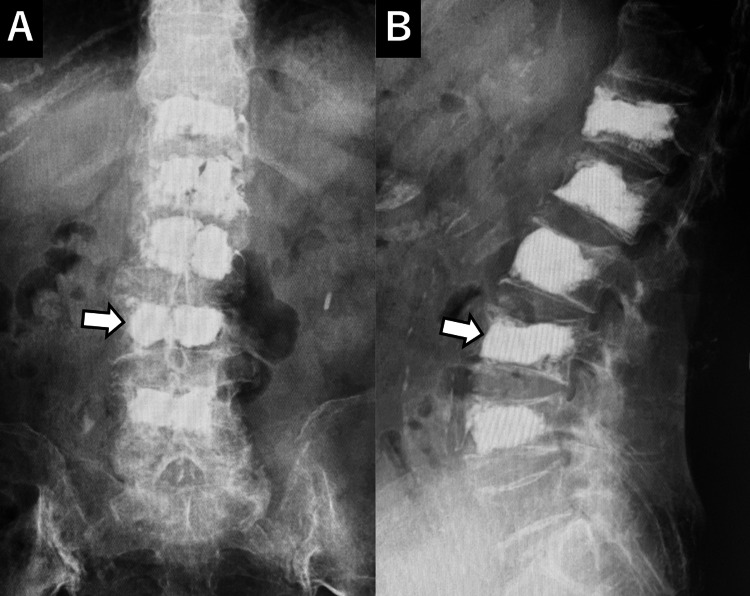
Post-third BKP X-ray An X-ray taken after the third balloon kyphoplasty (BKP) shows bone cement in L3 (arrow).

Although conservative treatment was attempted, it was ineffective. Given the patient's need to maintain independence, early surgical intervention was considered necessary. Therefore, a third percutaneous BKP was performed six weeks after the second surgery, with bone cement injected into the fractured L3 vertebra (Figure 7).

This procedure resulted in rapid pain relief, and no further fractures occurred during follow-up.

**Figure 8:**
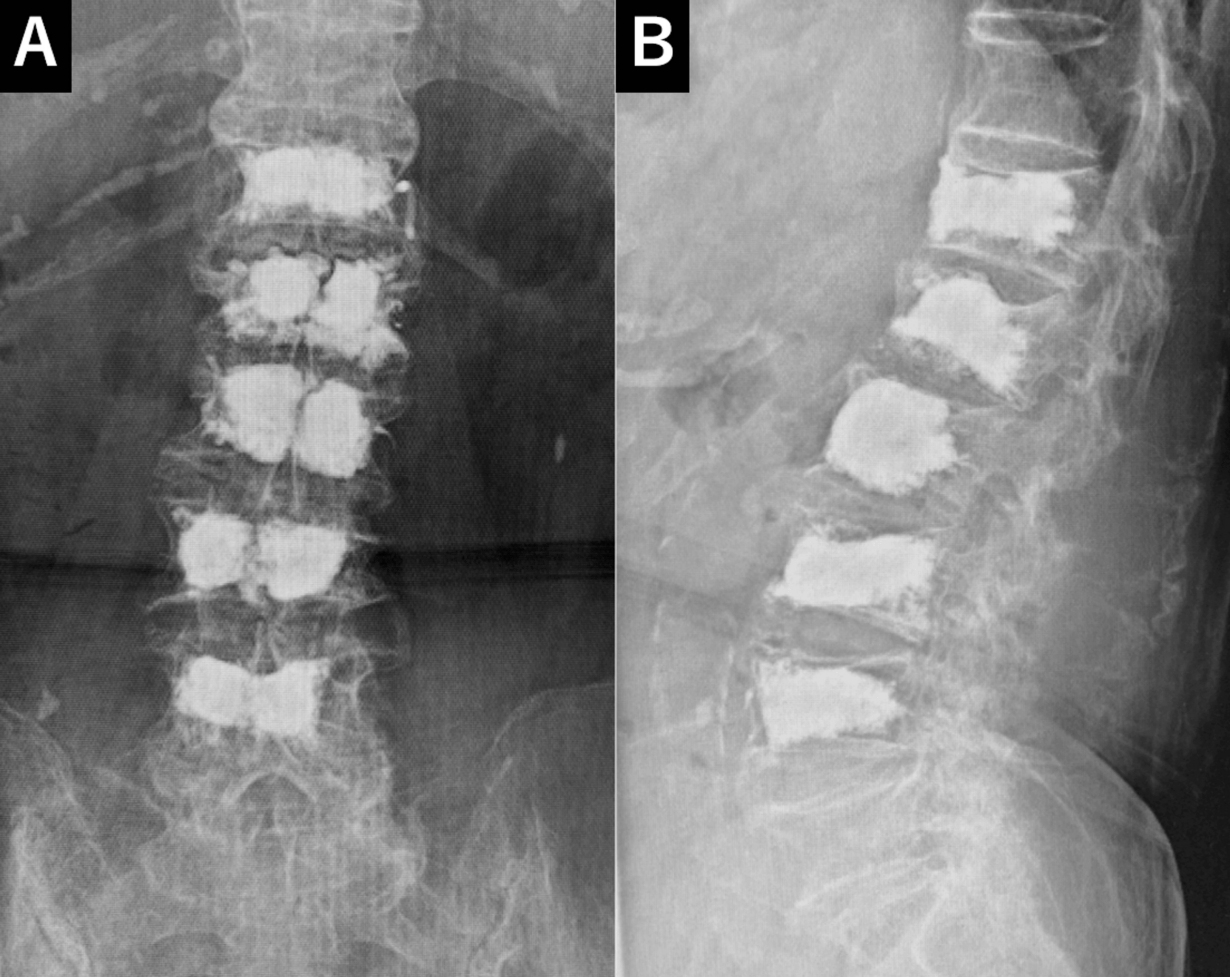
X-ray one year after BKP No new vertebral compression fractures are observed, and spinal alignment is maintained. BKP: Balloon kyphoplasty

In total, within four months from the initial fracture, the patient underwent BKP for five vertebral bodies (Th12, L1-L4). After the third surgery, her condition remained stable. She continued osteoporosis treatment with teriparatide and wore a corset for three months. Subsequent follow-up revealed no new fractures, and at one year post-surgery, X-rays showed good maintenance of vertebral body heights, with minimal progression of localized kyphosis (Figure 8).

Five years after the initial fracture, at the age of 92, no new vertebral fractures were observed, and the patient was able to walk independently without assistance, maintaining a high level of ADL.

## Discussion

This case presents an 87-year-old patient with osteoporosis-related vertebral fractures who successfully underwent repeated BKP, achieving pain relief and maintenance of ADL. BKP is an effective intervention for osteoporosis-related vertebral fractures that are resistant to conservative treatment. It is known to contribute to early pain relief, recovery of vertebral height, and inhibition of progression of spinal deformity [5,7,11]. Particularly in elderly patients, pain management and early mobilization are directly linked to prevention of disuse syndrome and facilitating social reintegration. Therefore, rapid intervention is of great significance. In this case, despite the patient's advanced age, early mobilization after each surgery was achieved, allowing continued independent living at home. It is presumed that the maintenance of vertebral height through BKP contributed to inhibiting the progression of spinal deformity and maintaining long-term ambulatory ability.

On the other hand, the incidence of adjacent vertebral fractures after BKP has been reported to range from 6.5% to 33%, with higher risks in women, the elderly, fractures at the thoracolumbar junction, and those with a history of vertebral fractures [6,9,10]. In this case, adjacent vertebral fractures occurred shortly after the initial BKP, which may have been influenced by the severe osteoporosis and local biomechanical changes. Additionally, the presence of multiple vertebral fractures at L1 and L4, and the use of bone cement for multiple vertebrae during the initial surgery, were risk factors. Given these factors, it was somewhat predictable that further fractures might occur at adjacent levels postoperatively; however, the occurrence of a fracture cascade affecting five vertebrae within a short period exceeded expectations and indicated the severity of osteoporosis in this patient.

The effects of osteoporosis treatment with teriparatide typically take 3-6 months to manifest, during which time bone fragility remains, making it difficult to prevent new vertebral fractures entirely [12-14]. Therefore, until the effects of bone metabolism improvement are achieved, it is crucial to employ appropriate interventions, such as external fixation with a corset or minimally invasive surgical interventions like BKP, to prevent ADL decline due to new vertebral fractures. Additionally, poor sagittal alignment in patients with osteoporosis-related vertebral compression fractures has been associated with sarcopenia and persistent low back pain [15], and it has been shown that PKP can improve sagittal alignment [16]. In this case, the timely intervention with BKP after each fracture helped minimize the development of local kyphotic deformity, allowing for long-term maintenance of spinal alignment and functional independence. Furthermore, BKP can be performed under general anesthesia in a short, minimally invasive procedure, making repeated interventions feasible even for elderly and frail patients. This case suggests that even in cases where multiple vertebral fractures occur in a short period, appropriate intervention at the right time can prevent immobility and maintain QOL.

Although this is a single case, and caution is needed when generalizing, it provides valuable insight into the potential of repeated surgical intervention using BKP as a bridging strategy until the effects of osteoporosis treatment take hold in elderly patients with severe osteoporosis.

## Conclusions

We encountered a case of a severely osteoporotic, super-aged patient who developed consecutive fractures of adjacent vertebral bodies within a short period, resulting in repeated BKP on five vertebrae. Early BKP intervention in each instance provided rapid pain relief and improved posture, allowing the patient to maintain independence in ADL over the long term. This case highlights the efficacy of BKP for osteoporotic vertebral fractures and underscores the importance of repeated minimally invasive interventions when the effects of osteoporosis treatment are delayed. Moving forward, it is essential to strike a balance between appropriate medical and surgical treatments for osteoporosis, with the goal of preserving the QOL of super-aged patients.
